# Mucosal candidiasis elicits NF-κB activation, proinflammatory gene expression and localized neutrophilia in zebrafish

**DOI:** 10.1242/dmm.012039

**Published:** 2013-05-29

**Authors:** Remi L. Gratacap, John F. Rawls, Robert T. Wheeler

**Affiliations:** 1 Department of Molecular and Biomedical Sciences, University of Maine, Orono, ME 04469, USA; 2 Department of Cell Biology and Physiology, University of North Carolina at Chapel Hill, Chapel Hill, NC 27599, USA; 3 Department of Microbiology and Immunology, University of North Carolina at Chapel Hill, Chapel Hill, NC 27599, USA; 4 Graduate School of Biomedical Sciences and Engineering, University of Maine, Orono, ME 04469, USA

## Abstract

The epithelium performs a balancing act at the interface between an animal and its environment to enable both pathogen killing and tolerance of commensal microorganisms. *Candida albicans* is a clinically important human commensal that colonizes all human mucosal surfaces, yet is largely prevented from causing mucosal infections in immunocompetent individuals. Despite the importance of understanding host-pathogen interactions at the epithelium, no immunocompetent vertebrate model has been used to visualize these dynamics non-invasively. Here we demonstrate important similarities between swimbladder candidiasis in the transparent zebrafish and mucosal infection at the mammalian epithelium. Specifically, in the zebrafish swimmbladder infection model, we show dimorphic fungal growth, both localized and tissue-wide epithelial NF-κB activation, induction of NF-κB -dependent proinflammatory genes, and strong neutrophilia. Consistent with density-dependence models of host response based primarily on tissue culture experiments, we show that only high-level infection provokes widespread activation of NF-κB in epithelial cells and induction of proinflammatory genes. Similar to what has been found using *in vitro* mammalian models, we find that epithelial NF-κB activation can occur at a distance from the immediate site of contact with epithelial cells. Taking advantage of the ability to non-invasively image infection and host signaling at high resolution, we also report that epithelial NF-κB activation is diminished when phagocytes control the infection. This is the first system to model host response to mucosal infection in the juvenile zebrafish, and offers unique opportunities to investigate the tripartite interactions of *C. albicans*, epithelium and immune cells in an intact host.

## INTRODUCTION

*Candida albicans* is a commensal fungus of human mucosa commonly found in the oropharynx, digestive system and female reproductive tract (Reef et al., 1998; Rindum et al., 1994; Scully et al., 1994; Soll et al., 1991). This opportunistic pathogen can produce both non-lethal localized mucosal and life-threatening systemic infections. Major advances in our molecular understanding of mucosal candidiasis have been achieved through combining *in vitro* and *in vivo* experiments (Naglik et al., 2008; Reef et al., 1998; Rindum et al., 1994; Scully et al., 1994; Soll et al., 1991), yet the spatiotemporal dynamics of this infection have proven difficult to dissect with existing experimental platforms.

Epithelial cells play an important role in signaling professional immune cells to mount an immune response to *C. albicans*. Although the receptors that activate epithelial cells are not all known (Cheng et al., 2012; Weindl et al., 2010), their engagement activates the NF-κB pathway in addition to other transcription factors (Moyes et al., 2010). This leads to induction of proinflammatory genes that recruit and activate professional immune cells to the site of infection (Moyes and Naglik, 2011). Neutrophils play an active role in enhancing epithelial immune response (Weindl et al., 2007), but are also associated with increased disease symptoms and immunopathology (Fidel et al., 2004; Lionakis et al., 2012). The *in vivo* mechanisms of neutrophil recruitment in mucosal candidiasis remain unclear, and might include chemokines, defensins and/or acute phase proteins such as serum amyloid A, all of which are highly upregulated in epithelial cells after infection with *C. albicans* (Conti et al., 2009; Tomalka et al., 2011).

The larval zebrafish (*Danio rerio*) is a practical and versatile model that offers unique experimental advantages over other systems, including the transparency of larvae and the lack of adaptive immune responses for the first few weeks after hatching (Tobin et al., 2012). Zebrafish models have been developed to study several diseases caused by human pathogens (Meijer and Spaink, 2011; Tobin et al., 2010), including systemic candidiasis (Brothers et al., 2011; Chao et al., 2010), but immune responses in a mucosal infection model have yet to be characterized in this transparent vertebrate host. Intriguingly, the only case descriptions of fish infections with *C. albicans* are mucosal infections of the swimbladder (Galuppi et al., 2001; Hatai, 1992).

The swimbladder shares functional, anatomical, ontological and transcriptional similarities to the lung. It is used for buoyancy, but maintains an air-mucosal interface that performs gas exchange to the circulatory system in some species (Lapennas and Schmidt-Nielsen, 1977). It develops from the foregut and remains connected to it through the pneumatic duct (Field et al., 2003), which is a potential infection route for ingested bacterial and fungal pathogens (Ross et al., 1975). Anatomically, the swimbladder epithelium is most similar to the lung epithelium, with a single layer of squamous epithelial cells covering the mesenchyme and a mesothelial layer (Robertson et al., 2007; Winata et al., 2009). It has a transcriptional signature that is very similar to the mammalian lung (Winata et al., 2009; Zheng et al., 2011) and has been shown to secrete both surfactant proteins (Sullivan et al., 1998) and β-defensin-like molecules (Oehlers et al., 2011a). This suggests that the swimbladder is a potentially useful organ for modeling other mucosal infections such as lung infections, in addition to being a natural site of infection for *C. albicans* in fish.

**TRANSLATIONAL IMPACT**

**Clinical issue**

*Candida albicans*, a fungus, is a ubiquitous human commensal that causes non-lethal mucosal infections at numerous sites, most frequently the vaginal and oropharyngeal tract and, in rarer cases, the lung and skin. The cost of care for candidiasis-associated vulvovaginal inflammation in the US exceeds $1.8 billion annually, with more than half of women estimated to experience at least one episode during their lifetime. The innate and adaptive arms of the immune system contribute to both protection and exacerbation of mucosal candidiasis, but our understanding of mucosal candidiasis has substantial gaps. Current experimental models of mucosal candidiasis focus on using immunocompromised murine and *in vitro* reconstituted epithelial systems to identify key mediators of immune response and fungal virulence. However, the complexity of dynamic interactions during infection demands a non-invasive model in which *C. albicans*, epithelial cells and immune cells can all be imaged in the context of normal three-dimensional tissue architecture in a fully immunocompetent host. Despite strong conservation of basic immune pathways from fish to human, there is currently no zebrafish model for host-pathogen interaction at the epithelium. Therefore, a transparent zebrafish model with facile genetic manipulation and intravital imaging offers an excellent platform for gaining insights in this disease.

**Results**

To generate a model for mucosal candidiasis, the authors infected the zebrafish swimbladder with *C. albicans*. In this mucosal infection model, *C. albicans* grows on the swimbladder epithelium as both yeast (unicellular fungi) and hyphae (long filamentous structures), as observed in mammalian infections *in vitro* and *in vivo*. The authors observed NF-κB activation in response to infection, occurring in a local or epithelial tissue-wide manner depending on the fungal burden. Global activation of NF-κB during high-level infection was shown to be accompanied by induction of two key pro-inflammatory genes, *saa* and *tnf*, that are also induced by *C. albicans* in mammalian epithelia. Similar to both oral and vulvovaginal candidiasis, neutrophils were found to be present at high numbers at the site of infection. Exploiting the ease of intravital imaging in zebrafish, the group also showed that phagocyte engulfment correlates with a decrease in NF-κB activation.

**Implications and future directions**

This study describes a new, tractable model of mucosal candidiasis and exploits its unique attributes to identify links between fungal location, immune response and epithelial response. The authors’ *in vivo* observations of differential transcription factor activation and gene expression as a function of fungal numbers confirm recent groundbreaking *in vitro* findings. The model developed here has important mechanistic resemblances to mucosal candidiasis in mammals. On the pathogen side, the model holds potential for elucidating the genetic requirements for virulence of *C. albicans*. On the host side, the mechanistic basis for signaling and phagocyte responses can be addressed using non-invasive imaging. This immunocompetent and transparent model also provides a unique tool for the study of cross-talk among epithelial cells and innate immune components in protecting against mucosal infection. In the future, this model could be extended to the study of more traditional respiratory pathogens such as mycobacteria and dimorphic fungi. Equally, this model could be used in genetic or chemical screens to identify novel mediators of epithelial immunity and virulence.

Here we use the transparent zebrafish swimbladder to model mucosal candidiasis. We show that this infection reproduces important aspects of fungal-epithelial interaction previously characterized *in vitro*, in murine models and in human disease. We find that high-level infection induces strong activation of NF-κB, transcriptional upregulation of NF-κB-dependent proinflammatory gene expression and robust neutrophilia. The strong neutrophil presence and associated engulfment of *C. albicans* at low-level infection might limit direct contact of yeast with epithelial cells, diminishing both NF-κB activity in these epithelial cells and expression of pro-inflammatory cytokines. The ability to follow both the host and pathogen non-invasively provides a powerful alternative model for understanding the molecular mechanisms underlying virulence and immunity in mucosal candidiasis.

## RESULTS

### *C. albicans* infects the zebrafish swimbladder and grows dimorphically

Mucosal candidiasis is the most common form of infection by *C. albicans* (Moran et al., 2012), but available *in vivo* animal models have limitations for imaging immune cell and pathogen interactions intravitally. We sought to exploit the transparency of the juvenile zebrafish to investigate the interactions of the epithelium, innate immune cells and pathogen during mucosal candidiasis.

We developed a non-invasive model of mucosal candidiasis of the swimbladder with infection through immersion. It is based on the premise that infection occurs naturally upon inflation of the swimbladder and the completion of the intestinal tract, at around 4 days post fertilization (dpf) (Kimmel et al., 1995; Pack et al., 1996). We found that immersion of zebrafish larvae, beginning at 3 dpf, with 4×10^7^ colony forming units (cfu)/ml of *C. albicans* gives the most consistent and highest infection rate.

**Fig. 1.**
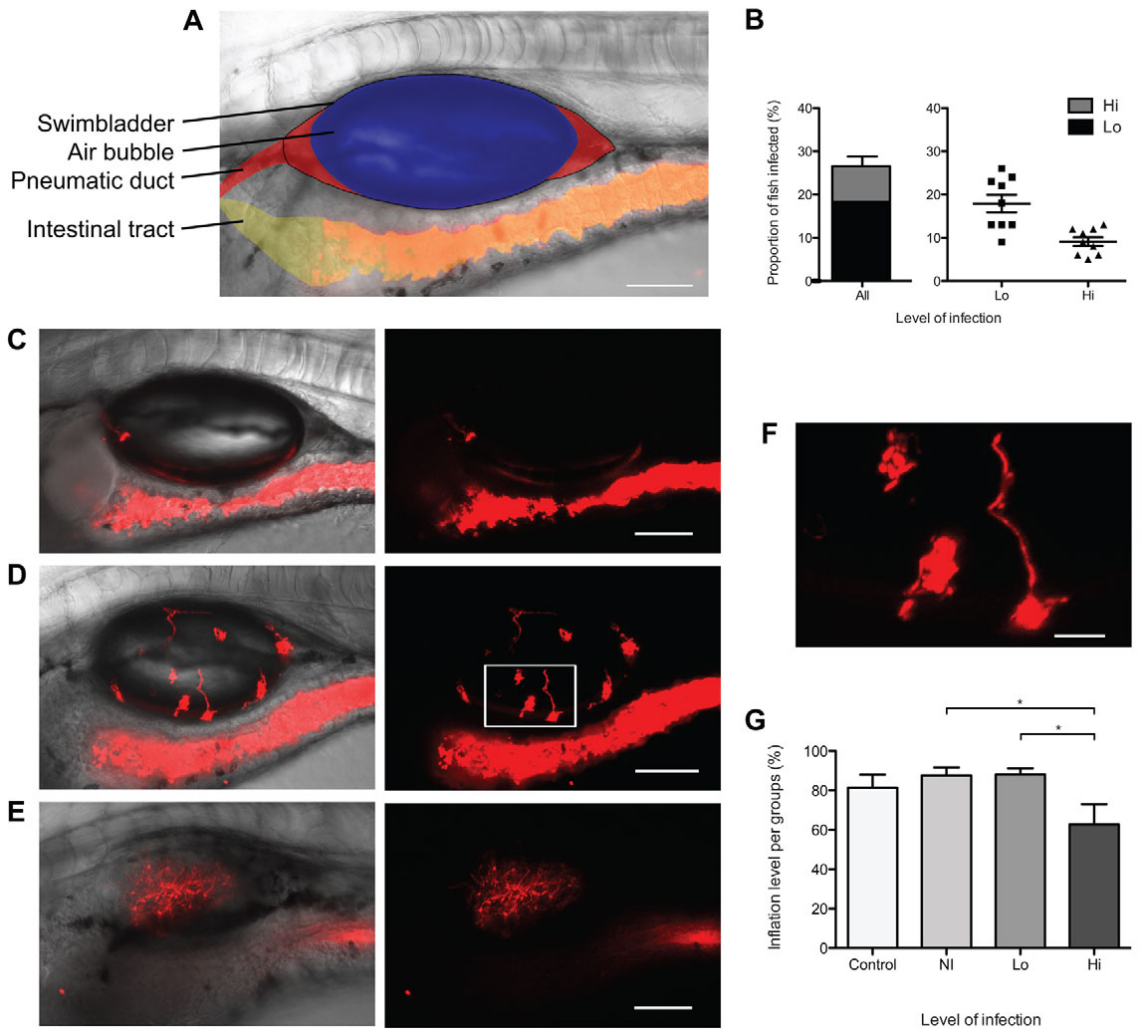
***Candida albicans* infects the swimbladder of juvenile zebrafish.** (A–F) Cohorts of 20 AB fish were infected by immersion with *C. albicans* CAF2-dTomato and imaged by confocal microscopy at 5 dpi (8 dpf). (A) *C. albicans* immersion, non-infected (NI) with pseudo-coloring; black outline of the swimbladder; blue: swimbladder air bubble; red: fluid filled regions, anterior with pneumatic duct and posterior; yellow: intestinal tract with red-fluorescent *C. albicans*. (B) Level of infection at 5 dpi, low-level infection (Lo; 1 to 20 yeasts), high-level infection (Hi; over 20 yeast cells) and combination of both. Left is a stacked chart depicting the overall percentage of infected fish, divided by intensity of infection. Right shows the mean and standard errors for nine independent experiments. (C–F) Representative images of different levels of infection after *C. albicans* immersion: (C) low-level infection; (D,E) high-level infection, with (D) inflated and (E) non-inflated swimbladder. Animated *z*-stack of panel D is shown in supplementary material Movie 1. (F) Magnification of panel D (white box). Scale bars: 100 μm (A,C–E) and 20 μm (F); maximum projection slices *n*=16 for all images. (G) Level of inflation of the swimbladder in different groups. Average and standard error of ten independent experiments are shown. One-way ANOVA and Bonferroni post-hoc test; *P<0.05. Data are representative of at least three independent experiments.

This immersion model results in highly reproducible infection of the swimbladder. At 2 days post-immersion (dpi), we found that the intestinal lumen is entirely filled with fluorescent yeast cells (Fig. 1A). Yeasts were occasionally seen within the pneumatic duct (data not shown), which remains open throughout the life of the zebrafish (Goolish and Okutake, 1999), suggesting that they enter the swimbladder through this route. By 5 dpi, ∼27% of fish had infected swimbladders (Fig. 1B), 18% with fewer than 20 yeast cells (low-level infection) and 9% with over 20 (high-level infection). At low-level infection, the majority of fungi remained as yeast (Fig. 1C) but hyphae occasionally germinated even at low burden. Germination within the swimbladder was more common in high-level infection, where the swimbladder could be observed filled with yeast and hyphae (Fig. 1D–F; supplementary material Movie 1). Although in >99% of cases fungal cells were only observed luminal to the apical surface of the epithelium, in rare cases hyphae pierced through the swimbladder and reached nearby tissue (supplementary material Fig. S1 and Movie 2). Yeasts were infrequently observed in organs outside the swimbladder and intestinal tract. *C. albicans* infection of the swimbladder is thus largely limited to the epithelium.

Because active infection is reflected in proliferation of fungi at the infection site, we performed infections with dTomato-expressing fungi that were also labeled with fluorescein isothiocyanate (FITC). This permits the distinction of the inoculum (dTomato-expressing and FITC-positive), new cells that grew within the fish (dTomato-expressing but FITC-negative) and cells from the inoculum that were killed (dTomato-negative but FITC-positive). These experiments show that both fungal cell division and killing of fungi occur during infection, suggesting that it is a dynamic process (supplementary material Fig. S2).

To assess whether only live yeasts can reach the swimbladder, we performed mock infections with heat-killed (HK) fungi. We found that HK yeast cells can enter the swimbladder at a similar level as live yeasts (supplementary material Fig. S3); however, high-level exposure of the swimbladder to HK fungi is probably a result of the increased clumping of these cells compared with live *C. albicans* (supplementary material Fig. S3A).

Although there were few gross phenotypic signs of infection through to 5 dpi, fewer fish with high-level infection had inflated swimbladders (Fig. 1G), suggesting that infection can disturb the normal inflation and/or maintenance of the air bubble. Although we followed survival of larvae beyond 5 dpi (8 dpf), the impact of infection on mortality could not be reliably quantified due to variability in mortality during late larval stages.

In this new model of mucosal candidiasis, we show that when *C. albicans* yeasts enter the swimbladder they can cause both lowand high-level infections, germinating and forming hyphae. The limited germination and proliferation of *C. albicans* on the epithelial surface is similar to colonization of immunocompetent mammals. The reproducible nature of these infections of the swimbladder in the transparent zebrafish enables non-invasive imaging of host-pathogen dynamics.

### NF-κB activity is enhanced *in vivo* during mucosal infection

*C. albicans* is actively recognized by epithelial cells, which results in activation of several signaling pathways both *in vitro* and *ex vivo* (Moyes et al., 2010). The NF-κB transcriptional pathway is an essential component in immune response to infection (Baeuerle and Henkel, 1994). Here, we exploited the swimbladder candidiasis model to study NF-κB activity *in vivo* using the *Tg(NFκB:EGFP)* transgenic fish line, in which NF-κB activity drives expression of enhanced green fluorescent protein (EGFP) (Kanther et al., 2011).

**Fig. 2.**
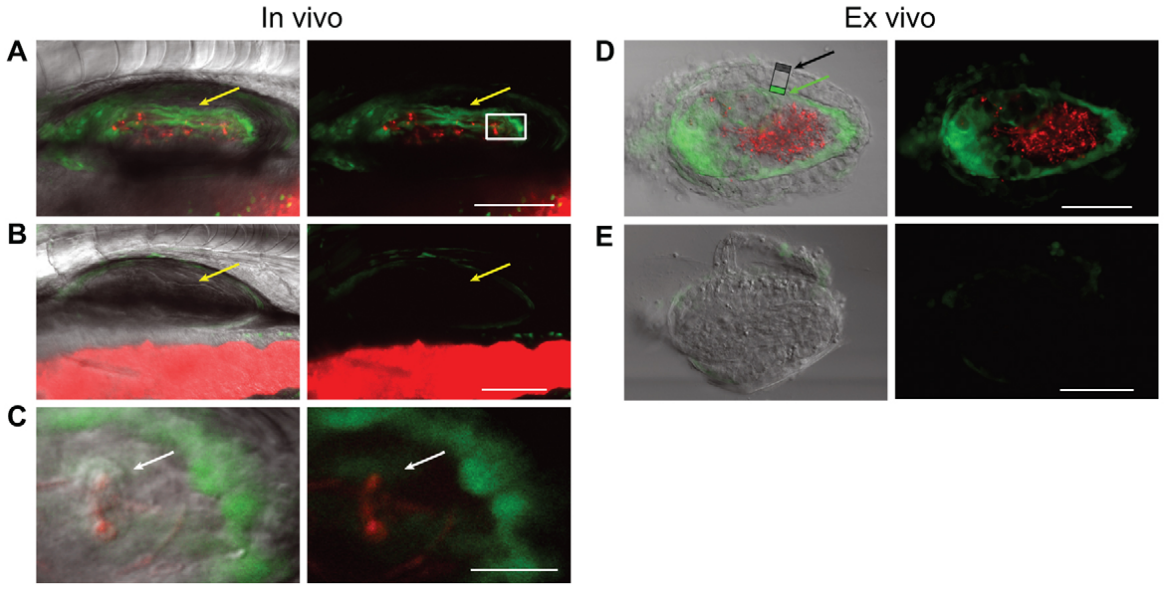
**NF-κB is highly activated in epithelial cells *in vivo* in high-level infection.** (A–E) Cohorts of twenty *NFκB:EGFP* fish were infected by immersion with *C. albicans* CAF2-dTomato and imaged by confocal microscopy at 5 dpi. (A–C) *In vivo* expression of GFP in high-level-infected (A) and uninfected (B) swimbladder; yellow arrows indicate the epithelial layer. (C) Magnification of image A (white box), with *C. albicans* inside a phagocyte (white arrows). (D,E) *Ex vivo* dissected swimbladder expression of EGFP from high-level infected zebrafish (D) with highlighted epithelial (green arrow towards green box) and mesothelial (black arrow towards gray box) layers and uninfected swimbladder (E). Scale bars: 100 μm (A,B,D,E) and 20 μm (C). Maximum projections of *n* slices: *n*=8 for A and B, *n*=1 for C–E. Images are representative of four independent experiments.

We found that infection of the swimbladder by *C. albicans* leads to NF-κB activation in epithelial cells. In high-level infection, the epithelium of the swimbladder expressed strong NF-κB-driven EGFP fluorescence (Fig. 2A,B). The fluorescence was widespread throughout the epithelial layer of the swimbladder, but was restricted to this layer (Fig. 2A–C). The magnitude of activation was more obvious *ex vivo*, where it is clear that the epithelial cell layer in infected swimbladders fluoresce more strongly than those in uninfected swimbladders (Fig. 2D,E). In swimbladders with high-level infection, phagocytes were often seen within the lumen, particularly in swimbladders in which the air bubble was not present. These phagocytes could harbor engulfed yeasts or pseudohyphae, and often expressed low-level EGFP fluorescence (Fig. 2C). Although mammalian phagocytes have been shown to activate NF-κB in response to pathogenic stimuli, expression of EGFP in this reporter line has not yet been explicitly defined (Kanther et al., 2011). Therefore, the lack of robust reporter gene expression might be due to limitations of the reporter rather than a lack of NF-κB activation. In uninfected swimbladders, there was weak fluorescence in the mesothelium and scattered EGFP-positive cells were present in the gut, as previously reported (Kanther et al., 2011). These results show for the first time that epithelial cells respond to mucosal *C. albicans* infection through NF-κB activation *in vivo*. Moreover, they demonstrate that the vast majority of the epithelial cells in the swimbladder are activated during high-level infection.

**Fig. 3.**
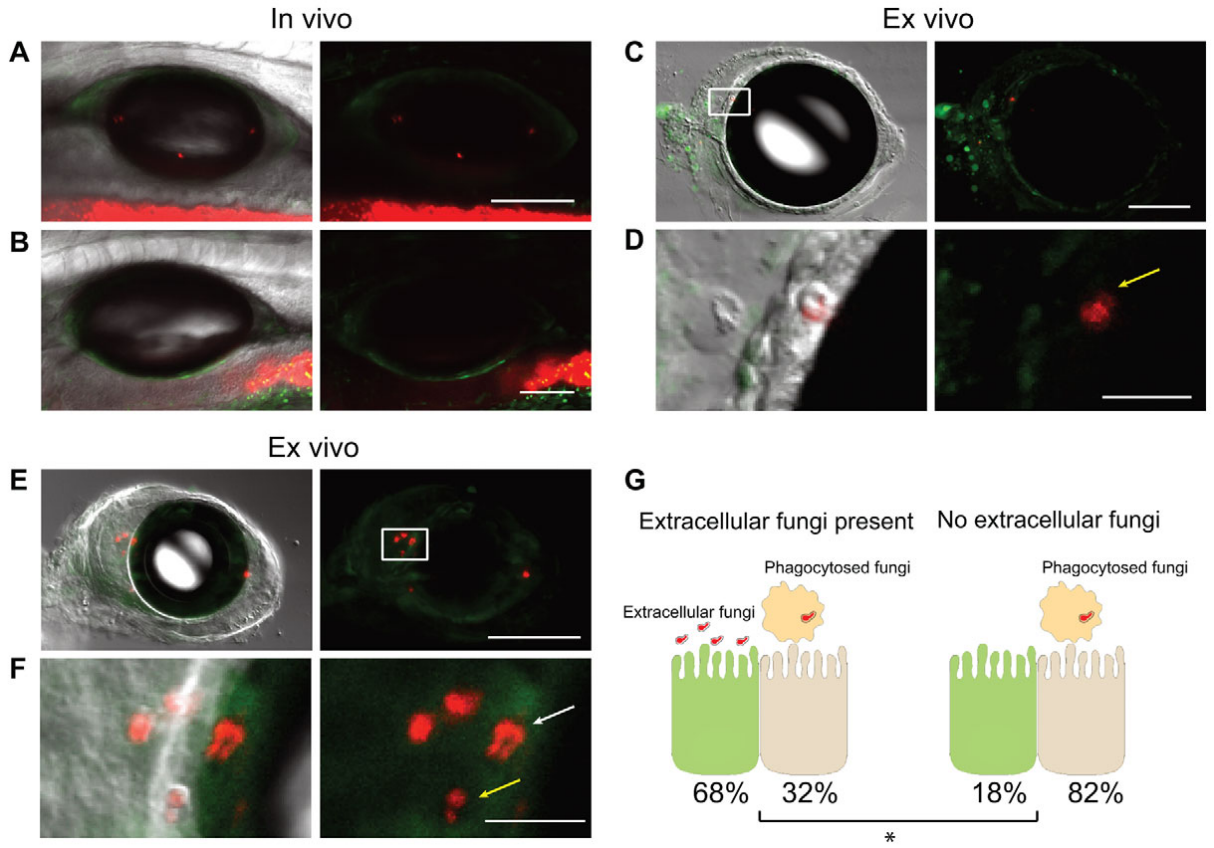
**NF-κB is activated *in vivo* in the swimbladder upon infection and activation is enhanced by interaction of *C. albicans* with the epithelium.** (A–F) Cohorts of 20 *NFκB:EGFP* fish were infected by immersion with *C. albicans* CAF2-dTomato and imaged by confocal microscopy at 5 dpi. (A,B) *In vivo* NF-κB activity in low-level-infected (A) and uninfected (B) swimbladder. (C–F) *Ex vivo* dissected swimbladder with low-level infection. (C) Fully inflated dissected swimbladder with (D) yeast cell inside a phagocyte (yellow arrow), and (E) partially inflated dissected swimbladder with (F) yeast cells in direct contact with epithelium (white arrow) and inside a phagocyte (yellow arrow). Animated *z*-stack of panel C is shown in supplementary material Movie 3, and zoomed *z*-stack is shown in supplementary material Movie 4. Animated *z*-stack of panel E is shown in supplementary material Movie 5, and zoomed *z*-stack is shown in supplementary material Movie 6. (D,F) Magnifications of C and E (white boxes). (G) Schematic representation of the epithelial response to the presence of extracellular fungi (E,F) or phagocytosed fungi only (C,D). Presence of extracellular *C. albicans* and epithelial cell EGFP (green) expression at 37 infection foci was quantified in images from 13 different fish in four independent experiments. There is a significantly higher proportion of cases of detectable EGFP in epithelial cells when there are extracellular fungi present. Fisher’s exact test; *P<0.05. Scale bars: 100 μm (A–C,E) and 20 μm (D,F). Maximum projections of *n* slices: *n*=11 (A), *n*=8 (B,C) and *n*=1 (D,F). Images are representative of four independent experiments.

In low-level infection, we found that NF-κB activation correlates with direct interaction of yeasts with epithelial cells. In infected fish with a low number of yeasts in the swimbladder, no overall differences in NF-κB activity were seen, as compared with uninfected fish (Fig. 3A,B). In most cases, the yeast cells were engulfed by phagocytic cells and, in contrast with high-level infection, epithelial cells did not express increased levels of EGFP (Fig. 3C,D; supplementary material Movies 3, 4). However, if the yeast cells were not contained within phagocytes, the epithelial cell in contact with the pathogen and the neighboring cells had increased fluorescence (Fig. 3F; supplementary material Movies 5, 6). When infection foci were categorized into situations in which extracellular fungi were either present (Fig. 3G, left) or absent (Fig. 3G, right), we saw a much greater likelihood for detectable epithelial EGFP expression when extracellular fungi are present (*P*<0.05). The activation of NF-κB in neighboring epithelial cells was also observed more often in cases in which the swimbladder was only partially inflated and fluid was present in between the air bubble and the epithelium (Fig. 3E). These data suggest that, at low pathogen density, an effective phagocyte response limits the activation of NF-κB in epithelial cells. However, at high-level infection, *C. albicans* is poorly contained and elicits a widespread activation of NF-κB in the epithelial layer lining the swimbladder.

### *serum amyloid A* and *tumor necrosis factor a* are induced in high-level infection

A large number of immune-related genes are induced by *C. albicans* infection at the epithelial level, including several cytokines, chemokines and antimicrobial peptides (Conti et al., 2009; Tomalka et al., 2011). Several of these inflammatory molecules are under NF-κB control (Moyes et al., 2010). To measure downstream effects of increased NF-κB activity due to mucosal infection in the zebrafish, we measured expression of three inflammatory genes shown to have some dependence on NF-κB in zebrafish and/or in mouse (Baeuerle and Henkel, 1994; Kanther et al., 2011): *serum amyloid A* (*saa*), *interleukin-1β* (*il1b*) and *tumor necrosis factor*, isoforms a and b (*tnfa* and *tnfb*).

**Fig. 4.**
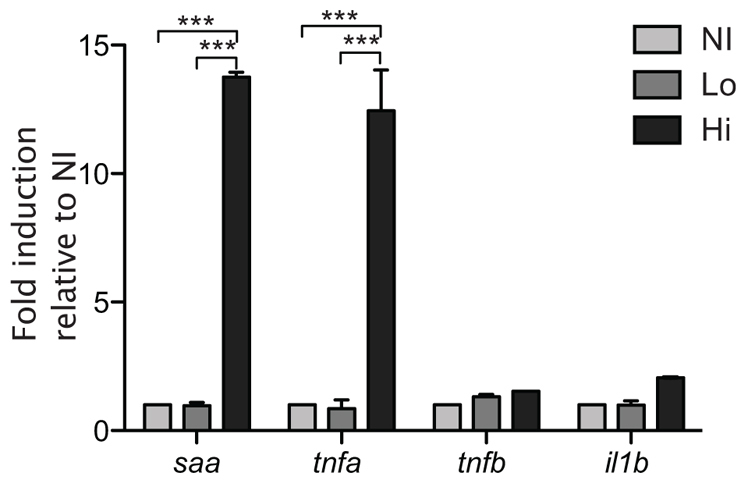
***saa* and *tnfa* are upregulated at high-level infection only.** Cohorts of 20 AB fish were infected by immersion with *C. albicans* CAF2-dTomato. At 5 dpi, fish were divided into groups according to infection level and homogenized for purification of total RNA. cDNA was synthesized and used for qPCR. Gene expression of *saa*, *il1b*, *tnfa* and *tnfb* in AB fish at 5 dpi was normalized to that of *gapdh* with non-infected (NI) used as the reference group (ΔΔCt), and expressed as fold induction (2^ΔΔCt^). Two-way ANOVA and Bonferroni post-hoc test; ***P<0.001. Means and standard errors shown are representative of five independent experiments. Lo, low-level infection; Hi, high-level infection.

Quantitative PCR with whole fish revealed that only expression of *saa* and *tnfa* were changed upon infection (Fig. 4). Comparisons with uninfected fish show that fish with high-level infection had increased expression of both *saa* and *tnfa*. This was not the case for fish with low-level infection, which had unchanged expression of all cytokines measured. Surprisingly, neither *il1b* nor *tnfb* expression was induced at any level of infection. However, the use of total RNA from whole animals could mask local tissue-specific expression differences. Notably, exposure of the swimbladder to HK yeast cells induced expression of the same genes as did live *C. albicans* (supplementary material Fig. S3D).

In summary, the gene expression data shows that some NF-κB-driven inflammatory genes are upregulated upon *C. albicans* infection in the zebrafish model presented here, similar to mucosal candidiasis in mammals (Dongari-Bagtzoglou and Fidel, 2005).

### Infection induces localized neutrophilia in proportion to fungal burden

Rapid recruitment of neutrophils to the site of infection is one of the hallmarks of the acute inflammatory response (Sohnle et al., 1976). NF-κB is an important activator of the mucosal immune response, which directs recruitment of neutrophils to the epithelium (Everhart et al., 2006; Mizgerd, 2002; Pantano et al., 2008). The recruitment and activation of these myeloid cells is mediated by chemokines and cytokines, among which TNF and SAA are important in response to *C. albicans* infection (Badolato et al., 2000; Netea et al., 1999; Weindl et al., 2007). To investigate the neutrophil immune response in swimbladder infection, we utilized *mpx:GFP* transgenic zebrafish (Renshaw et al., 2006), which have GFP-expressing neutrophils.

**Fig. 5.**
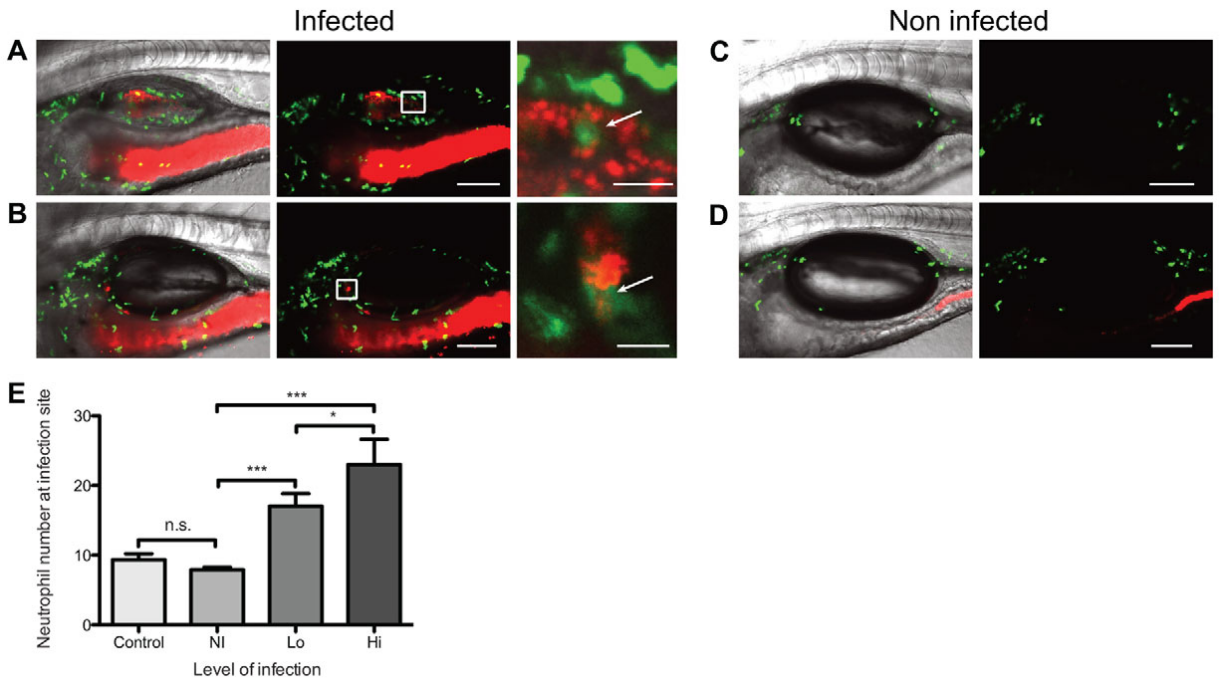
**Neutrophils are present at increased levels in swimbladder infection.** (A–D) Cohorts of 20 *mpx:GFP* fish were infected by immersion with *C. albicans* CAF2-dTomato and imaged by confocal microscopy at 5 dpi. (A,B) Neutrophil accumulation at the site of infection. (A) High-level infection in non-inflated swimbladder; (B) low-level infection in fully inflated swimbladder. Panel on the right is a magnification of red and green channels (white box) showing direct contact between neutrophil and *C. albicans* (arrows). Animated *z*-stack of panel A, right, is shown in supplementary material Movie 8. Animated *z*-stack of panel B, right, is shown in supplementary material Movie 7. (C,D) Non-infected control fish. (C) *C. albicans* was not added to the media and (D) *C. albicans* immersion, no infection. (E) Number of neutrophils in the swimbladder per individual fish. Average and standard error of three independent experiments are shown (pooled data). One-way ANOVA and Bonferroni post-hoc test; ***P<0.001; *P<0.05; n.s. non significant. Scale bars: 100 μm (left panel) and 20 μm (right panel) for magnification. Maximum projections of *n* slices: *n*=7 (A–D) and *n*=5 for magnification of A and B. Images are representative of four independent experiments.

We observed strong neutrophilia in the swimbladder in all levels of infection. More neutrophils were present in the swimbladder both in high- and low-level infection (Fig. 5A,B), as compared with the uninfected swimbladder (Fig. 5C,D). In addition, morphometric analysis of confocal *z*-stacks revealed that swimbladders with high-level infection had significantly more neutrophils than swimbladders with low-level infection and that both these are significantly different from non-infected groups (Fig. 5E). In low-level infection, neutrophils were found at infection foci and could be seen in direct contact with yeast cells (Fig. 5B; supplementary material Movie 7). In high-level infection, neutrophils were spread throughout the tissue surrounding the swimbladder and they could also be in direct contact with fungi (Fig. 5A; supplementary material Movie 8). Neutrophils were present at similar levels when the swimbladder was exposed to HK *C. albicans* (supplementary material Fig. S3C). These data show that *C. albicans* swimbladder infection is associated with an increased neutrophil response at the site of infection, even more so at high-level infection.

## DISCUSSION

We describe here a powerful new zebrafish model for the study of mucosal candidiasis. This extends previous models of invertebrate and rodent mucosal fungal infection (Naglik et al., 2008) into a transparent vertebrate that is reliant on the innate immune system. This zebrafish model shares key aspects of mucosal infection of the human and mouse, including dimorphic fungal growth, epithelial NF-κB activation, induction of NF-κB-dependent proinflammatory genes and localized neutrophilia. Exploiting unique advantages of the system, we non-invasively document a newly identified relationship between fungal burden and epithelial NF-κB activity as a function of phagocyte activity. We provide the first *in vivo* support for a proposed density-dependent model of host response based on *in vitro* results (Moyes et al., 2010). The amenability of the zebrafish platform for chemical and genetic screening provides a unique opportunity for the discovery of mechanisms underlying host response and fungal virulence.

This is the first reported zebrafish model of innate immune responses to epithelial infection with a human pathogen, and the first description of experimental swimbladder infection in fish larvae. It builds on previous work that has used the zebrafish to elucidate bacterial-intestinal interactions and host mechanisms responsible for gut development and immunity to intestinal pathogens (Flores et al., 2010; Hall et al., 2007; Kanther et al., 2011; O’Toole et al., 2004; Oehlers et al., 2011b; Pressley et al., 2005; Rendueles et al., 2012). In contrast to these studies, we model mucosal immunity to fungal infection in the swimbladder and focus on the cellular and molecular components of a successful and protective innate immune response to a human pathogen. The development of a mucosal model of candidiasis in a transparent vertebrate is especially important because it enables the study of intravital interactions of the pathogen with epithelial and innate immune cells. Striking similarities between the swimbladder and the mammalian lung reinforce the potential for this model to shed light on immune mechanisms involved in pulmonary fungal infection (Bals and Hiemstra, 2004; Cardoso and Lü, 2006; Daniels and Skinner, 1994; Field et al., 2003; Perrin et al., 1999; Prem et al., 2000; Rock and Hogan, 2011; Winata et al., 2009; Zheng et al., 2011). Although not typically regarded as a lung pathogen, recent work suggests that *C. albicans* might cause and/or exacerbate pulmonary infections (Leclair and Hogan, 2010; Ogba et al., 2013).

We exploited the advantages of this new model of mucosal candidiasis to demonstrate localized NF-κB activation by *C. albicans in vivo*, finding that it can occur at all infection levels. This is consistent with *in vitro* evidence that NF-κB is activated in epithelial cells by both high and low levels of *C. albicans* infection (Moyes et al., 2010; Steubesand et al., 2009). It is also consistent with evidence that NF-κB plays a protective role in candidiasis (Li and Dongari-Bagtzoglou, 2009; Moyes et al., 2010; Pivarcsi et al., 2003; Steubesand et al., 2009). In high-level swimbladder infection, epithelial cells exhibit widespread and strong NF-κB activity, as seen in lung epithelium after prolonged lipopolysaccharide infusion (Everhart et al., 2006). This suggests that high-level infections represent a state in which the epithelial cells are being continuously stimulated. In contrast, NF-κB signaling during low-level infection is apparently only activated by close-range signals when *C. albicans* is not contained within phagocytes. *In vitro C. albicans* epithelial infections suggest that NF-κB could be activated either through triggering of receptors by fungal products (Zhu et al., 2012; Zipfel et al., 2011) or through host-derived molecules activating neighboring bystander cells (Dongari-Bagtzoglou et al., 2004; Steubesand et al., 2009). This activation might also be accomplished by direct cell-cell activation through gap junctions, as has recently been shown for bacterial infection (Kasper et al., 2010). Our finding that only non-engulfed yeasts efficiently activate NF-κB suggests that fungal products might be required to mediate the activation of close-range bystander epithelial cells, but this does not rule out the participation of host molecules. The combination of *C. albicans* mutants and the versatile zebrafish toolbox offer unique access to further probe the mechanistic details of NF-κB activation during *in vivo* infection.

Using non-invasive phenotypic screening to correlate infection level with inflammation, we have identified conserved gene expression responses to mucosal candidiasis. The dependence of inflammatory gene expression on high-level infection is consistent with *in vitro* data that shows a density dependence of some inflammatory responses (Moyes et al., 2010; Steubesand et al., 2009). Low-level swimbladder infection elicits weak NF-κB activation and no significant overall activation of *saa* or *tnf*, whereas high-level infection strongly activates NF-κB and stimulates both *saa* and *tnf* transcription. Consistent with these results, colonization with commensal microbiota has been shown to upregulate *saa* in the intestine, swimbladder and liver in an NF-κB-dependent fashion (Kanther et al., 2011). The upregulation of *Saa* is also seen in an immunocompromised mouse model of oral candidiasis, and has been suggested to play a role in Th17 activation (Ather et al., 2011; Conti et al., 2009; Ivanov et al., 2009; Migita et al., 2010). TNF is highly upregulated by *C. albicans* and mediates protection against mucosal candidiasis (Farah et al., 2006; Weindl et al., 2007), as well as maintenance of the epithelial barrier (Eyerich et al., 2011). However, recent work suggests that TNF might play somewhat different roles in mammals and fish (Roca et al., 2008). Existing morpholino tools in the zebrafish system can be used to elucidate the precise function(s) of Saa and Tnf in protection against mucosal candidiasis *in vivo*.

The strong recruitment and/or retention of neutrophils in the infected swimbladder is consistent with what is seen in mammalian mucosal candidiasis (Challacombe, 1994; Sohnle et al., 1976). The strongest recruitment is seen during high-level swimbladder infection, when both *saa* and *tnf* are highly expressed. Interestingly, TNF upregulates chemotaxis through multiple mechanisms (Amulic et al., 2012) and SAA is a potent chemoattractant that might mediate chemotaxis towards *C. albicans* through the formylated peptide receptor (Edens et al., 1999; Su et al., 1999). However, the finding that neutrophils are recruited to the swimbladder even during low-level infection, in which there is weak NF-κB activity and no overall upregulation of *saa* or *tnf*, suggests that multiple pathways are responsible for the recruitment and retention of neutrophils at the infection site. Neutrophils are important in protection against mucosal candidiasis in humans and in animal models, but can also exacerbate symptoms (Akova et al., 1994; Anaissie and Bodey, 1990; Fidel et al., 2004). Their protective role has been established both through neutrophil ablation (Farah et al., 2001) and by IL-17 pathway disturbance (Conti et al., 2009). However, how neutrophils collaborate with epithelial cells is still unclear. Recently, several studies have highlighted a complementary role of neutrophils in enhancement of the expression of receptors and antimicrobial defenses on epithelial cells in mucosal candidiasis (Schaller et al., 2004; Steubesand et al., 2009; Weindl et al., 2007; Weindl et al., 2011). The zebrafish provides a unique model to investigate neutrophil recruitment, using a combination of gene knock-down or chemical inhibitors and time-lapse imaging. The functional relevance of neutrophils in immune-epithelial cross-talk as well as immunopathology (Lionakis et al., 2012; Wheeler et al., 2008) is now readily testable using a recently described conditional ablation transgenic fish line (Gray et al., 2011).

Our findings that HK yeast can elicit both increased pro-inflammatory gene expression and swimbladder neutrophilia suggest that at least some immune responses are due to direct effects of fungal recognition. This is consistent with numerous *in vitro* studies that have shown that HK *C. albicans* elicits strong immune responses from phagocytes (Jeremias et al., 1991). This also suggests that some immune responses at the epithelium can result from direct recognition of fungi through pattern recognition receptors, independent of the ability of *C. albicans* to grow invasively. Work in reconstituted human epithelial models has shown that HK hyphae can induce MKP1 expression (Moyes et al., 2010), although HK yeast fail to induce MKP1 or elicit immune responses (Moyes et al., 2010; Schaller et al., 2002; Schaller et al., 2005). Identification of the drivers of both shared and divergent responses in the swimbladder and reconstituted human epithelium models could shed light on conserved mechanisms of immune responses to mucosal candidiasis.

The development of the first model of fungal epithelial infection in the tractable larval zebrafish system opens up new possibilities in modeling other human mucosal pathogens. It also enables testing of a new set of hypotheses using real-time imaging of both *C. albicans* pathogenesis and host immune responses during mucosal infection in an intact vertebrate host.

## MATERIALS AND METHODS

### Zebrafish care and maintenance

All zebrafish were kept in recirculating systems (Aquatic Habitats) at the University of Maine Zebrafish Facility, under a 14/10 hour light/dark cycle. Water temperature was kept at 28°C. All zebrafish care protocols and experiments were performed in accordance with NIH guidelines under Institutional Animal Care and Use Committee (IACUC) protocol A2009-11-01. Larvae were rinsed in 0.15% Perosan solution (v/v in E3 media) for 1 minute after collection (Phennicie et al., 2010) and kept in a 28°C incubator at 80 fish per 50 ml in E3 media plus 0.00003% methylene blue for 24 hours and E3 media plus PTU (1-phenyl-2-thiourea, Sigma) thereafter (Brand et al., 2002). A concentration of 15 μg/ml PTU is sufficient to inhibit melanization and allows confocal imaging without impacting mortality or development; this is consistent with research by others (Karlsson et al., 2001) and was routinely confirmed in our experiments by noting survival and gross anatomical defects up to 8 dpf. Fish were fed to 0.01% w/v with dry food (ZM-000) daily from 6 dpf. The fish lines used were AB from ZIRC, *Tg(BACmpo:gfp)^114^* as described (Renshaw et al., 2006) and referred to as *mpx:GFP* hereafter and *Tg(NFκB:EGFP)^nc1^* as described (Kanther et al., 2011). When using *Tg(NFκB:EGFP)*, transgenic males were crossed with AB females. All zebrafish care and husbandry procedures were performed as described previously (Westerfield, 2000).

### Engineering of *C. albicans* fluorescent strains

The CAF2.1-dTom-NATr strain of *C. albicans* (CAF2-dTomato) was constructed by transforming CAF2.1 strain (Δ*ura3::imm434/URA3*) (Fonzi and Irwin, 1993) with the pENO1-dTom-NATr plasmid (supplementary material Fig. S4). This plasmid contains a codon-optimized version of the dTomato gene under the control of the constitutive *ENO1* promoter, with the nourseothricin resistance (NAT^r^) selection marker (pUC57 backbone, Genscript, Germany). The transformation was carried out with lithium acetate as previously published (Gietz et al., 1995), using nourseothricin resistance as an integration marker (100 μg/ml NAT, Werner Bioagents). Twenty colonies were selected and screened for fluorescence by flow cytometry (488/585 nm, FACScalibur, Becton Dickinson). A PCR check for integration was performed using the following primers to verify for correct plasmid integration (1185 bp): pENO1 FW: 5′-TCCTTGGCTGGCACTGAACTCG-3′ and dTom REV: 5′-AAGGTCTACCTTCACCTTCACC-3′.

### Fungal strains and growth conditions

CAF2-dTomato was grown on yeast-peptone-dextrose (YPD) agar. For infections, liquid cultures of *C. albicans* were grown overnight in YPD at 30°C on a roller-drum (New Brunswick Scientific). Overnight cultures were washed twice in phosphate-buffered saline (PBS) and the concentration was adjusted to 4×10^8^ cfu/ml. For preparation of HK fungi, CAF2-dTomato was grown in YPD overnight as previously described and the concentration adjusted to 3.2×10^8^ cfu/ml in PBS. HK yeasts were prepared by incubating in a boiling water bath for 15 minutes. HK and live yeasts were centrifuged and resuspended in 100 μl of PBS with 11 μl of Na_2_CO_3_ (1 M, pH 10) and 1 μl of Alexa Fluor 647 (Invitrogen, succinimidyl ester, 10 mg/ml in DMSO) or 1 μl of FITC (Invitrogen, 100 mg/ml in DMF). HK and live yeasts were incubated in the dark for 1 hour and vortexed every 15 minutes. The cells were then washed four times in PBS, resuspended in 1 ml of PBS at a concentration of 3.2×10^8^ cfu/ml and added to the E3 media as described.

### Bath infection

At 3 dpf, embryos were divided in groups of 20 into 15-ml conical tubes (Falcon, Becton Dickinson) containing 8 ml of E3 media plus PTU. CAF2-dTomato was added to each tube to the appropriate concentration. The tubes were placed in a roller-drum (40 rpm) in a 28°C incubator for the duration of the experiment in order to keep *C. albicans* in suspension. Media was changed daily (100% of the volume), the fish counted and reinfected immediately at the appropriate concentration.

### Fluorescence microscopy

For live imaging, fish were anesthetized in Tris-buffered Tricaine (200 μg/ml, Western Chemicals) and further immobilized in a solution of 0.4% low-melting-point agarose (LMA, Lonza) in E3 + Tricaine in a 24-well plate glass-bottom imaging dish (MatTek Corporation). For dissected swimbladders, fish were euthanized by an overdose of Tricaine and the swimbladder removed with dissection tweezers (#5, Electron Microscopy Sciences). Each dissected swimbladder was immediately placed in an imaging dish with 0.4% LMA and imaged within 10 minutes. Confocal imaging was carried out using an Olympus IX-81 inverted microscope with an FV-1000 laser scanning confocal system (Olympus). Objective lenses with powers of 4×/0.16 numerical aperture (NA), 10×/0.4 NA and 20×/0.7 NA (Olympus) were used. The EGFP and FITC, dTomato fluorescent protein, and Alexa Fluor 647 were detected by laser/optical filters for excitation/emission at 488/510 nm, 543/618 nm and 635/668 nm, respectively. Images were collected and processed using Fluoview (Olympus) and Photoshop (Adobe Systems Inc.). Panels are either a single slice for the differential interference contrast channel (DIC) with maximum projection overlays of fluorescence image channels (red-green), or maximum projection overlays of fluorescence channels. The number of slices for each maximum projection is specified as *n* in the legends of individual figures.

### RNA isolation and qPCR

Zebrafish infected with CAF2-dTomato were screened at 5 dpi by confocal microscopy and grouped as control (no *C. albicans* immersion), NI (CAF2-dTomato immersion, no infection), Lo (CAF2-dTomato immersion, 1–20 fungal cells in the swimbladder) and Hi (CAF2-dTomato immersion, over 20 fungal cells in the swimbladder). The division between Lo and Hi was chosen to be 20 fungi because this provides a relatively high upper limit of fungal burden in the Lo class, in which there is a more limited immune response. Total RNA was isolated from 20 whole larvae using a combination of Trizol (Invitrogen) and RNeasy column (Qiagen). Briefly, the Trizol isolation protocol was followed and the aqueous phase containing RNA was transferred to an RNeasy column following the manufacturer’s protocol for clean-up of RNA samples. Total RNA was eluted in 20 μl of nuclease-free water and stored at −80°C. cDNA was synthesized from 400 ng of tRNA with Improm-II kit (Promega), and a no-RT reaction was carried out for each sample. qPCR primers used in this study are shown in Table 1. A CFX96 thermocycler (Bio-Rad) was used with the following conditions: 95°C for 3 minutes, followed by 40 cycles of 95°C for 10 seconds, 57°C for 10 seconds and 72°C for 30 seconds; the final step included a dissociation curve. Threshold cycles (Ct) and dissociation curve were analyzed with Bio-Rad CFX Manager software. Gene expression levels were normalized to zebrafish *gapdh* (ΔCt) and compared to the NI group (ΔΔCt). Fold induction (2^ΔΔCt^) is represented.

**Table 1.**
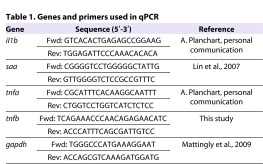
Genes and primers used in qPCR

### Statistics

Student’s *t*-test (two tailed, equal variance) and one/two-way ANOVA (plus Bonferroni post-hoc test for multiple comparison) was carried out using Prism5 (Graphpad Software Inc.) and *P*-values were considered significant for *P*<0.05 (*) and *P*<0.001 (***).

## Supplementary Material

Supplementary Material
