# Supplementary material for: Mucosal candidiasis elicits NF-κB activation, proinflammatory gene expression and localized neutrophilia in zebrafish
Source: Dis Model Mech. 2013 May 29;6(5):1260–70. doi: 10.1242/dmm.012039 (PMC3759345; doi:10.1242/dmm.012039)
Supplement: Supplementary Material [file supp_6_5_1260__index.html]

Mucosal candidiasis elicits NF-κB activation, proinflammatory gene expression and localized neutrophilia in zebrafish — Mucosal candidiasis elicits NF-κB activation, proinflammatory gene expression and localized neutrophilia in zebrafish — Supplementary Material 

# Mucosal candidiasis elicits NF-κB activation, proinflammatory gene expression and localized neutrophilia in zebrafish

## 

**Files in this Data Supplement:**

- **Supplementary Material PDF**
