# Supplementary material for: Mucosal candidiasis elicits NF-κB activation, proinflammatory gene expression and localized neutrophilia in zebrafish
Source: Dis Model Mech. 2013 May 29;6(5):1260–70. doi: 10.1242/dmm.012039 (PMC3759345; doi:10.1242/dmm.012039)
Supplement: Supplementary Material [file supp_012039_DMM012039.pdf]

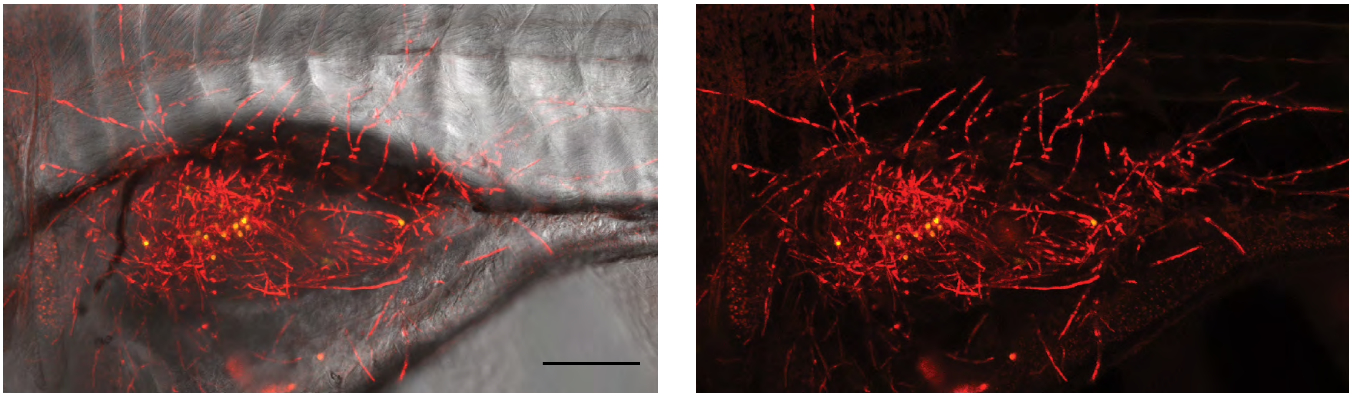

Fig. S1. Candida albicans germinates from the swimbladder and invades nearby tissues. AB fish were infected by immersion with FITC-labeled CAF2-dTomato C. albicans and imaged by confocal microscopy at 5 dpi. Filaments derived from FITC-labeled inoculating yeast are dTomato-expressing but not FITC labeled, as new cell wall does not inherit the FITC fluorescence and only the inoculated yeast are green. Scale bar represents 100 µm, maximum projection *n*=12. An animated Z-stack is shown in supplementary material Movie 8.

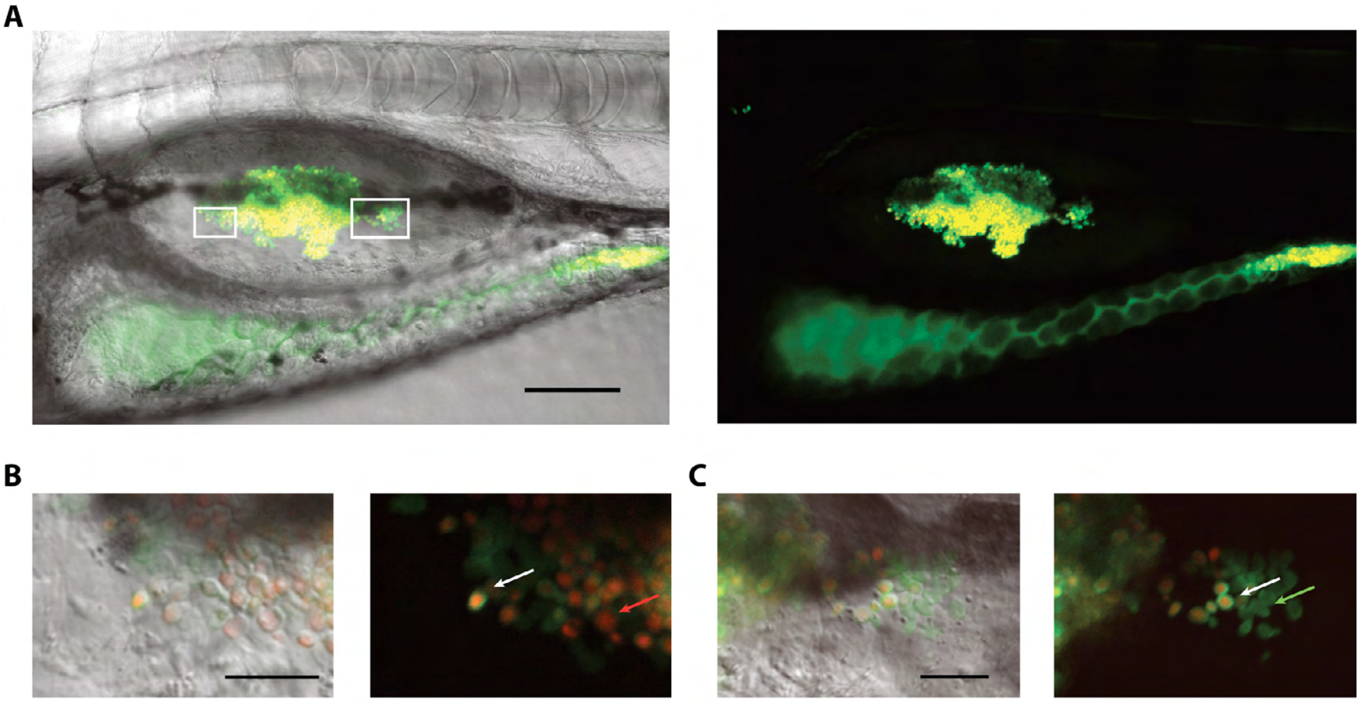

Fig. S2. C. albicans can both proliferate and be killed in the swimbladder. (A-C) AB fish were infected by immersion with FITC-labeled C. albicans and imaged by confocal microscopy at 5 dpi. Live cells from the inoculum are red in the cytosol and green in the cell wall, dead cells are green only and newly divided cells are red only. (A) *In vivo* high-level infection. (B) Magnification of A (left box) with live cells from the inoculum (white arrow) and newly divided cells (red arrow). (C) Magnification of A (right box) with live cell (white arrow) and dead cells (green arrow). Scale bars represent 100 µm (A) and 10 µm (B and C), maximum projection *n*=14 for A and *n*=1 for B and C. Images are representative of 3 independent experiments.

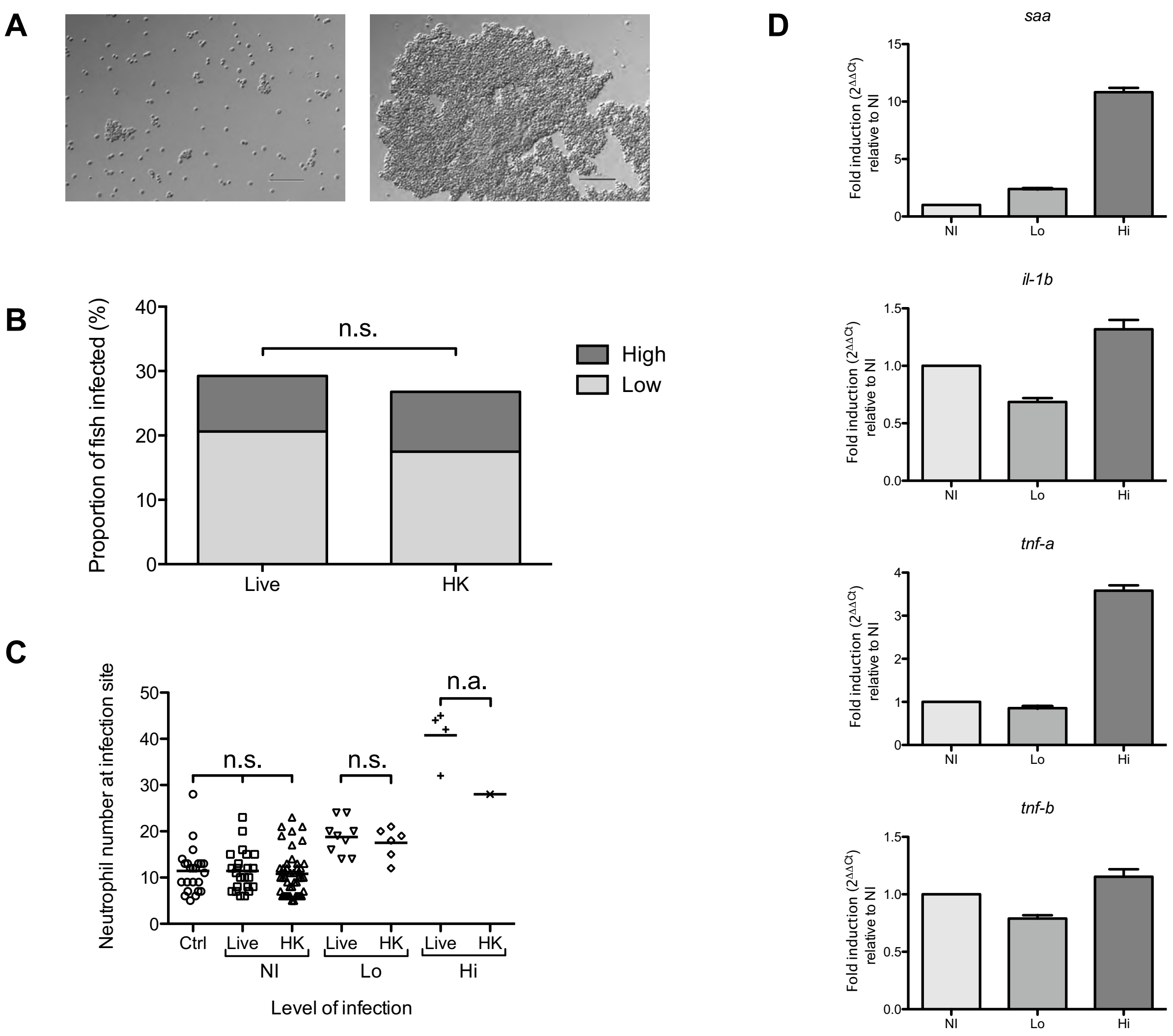

Fig. S3. Heat-killed C. albicans can enter the swimbladder and induce gene expression. (A) Image of live and heat-killed (HK) C. albicans in E3 media, collected after incubation for 24h with zebrafish; scale bars represent 50 µm. (B-D) Cohorts of zebrafish were infected by immersion with live or heat-killed CAF2-dTomato C. albicans. (B) Level of infection at 5 dpi with FITC-labeled live or HK C. albicans. Means of 4 independent experiments are shown. Two-way ANOVA and Bonferroni post-hoc test; n.s. non significant. (C) Number of neutrophils in the swimbladder of mpx:GFP fish per individual fish at 5 dpi with live or HK C. albicans fluorescently labeled with Alexa-647 to visualize HK cells. Data from two independent experiments were pooled, and means of the pooled data are represented. One-way ANOVA and Bonferroni post-hoc test; n.s. non significant; n.a. non applicable. (D) Gene expression of saa, il1b, tnfa and tnfb in AB fish at 5 dpi was normalized to that of gapdh and expressed as fold induction relative to the NI control group ($2^{\Delta\Delta Ct}$). Means and standard errors are representative of at least two independent experiments.

A

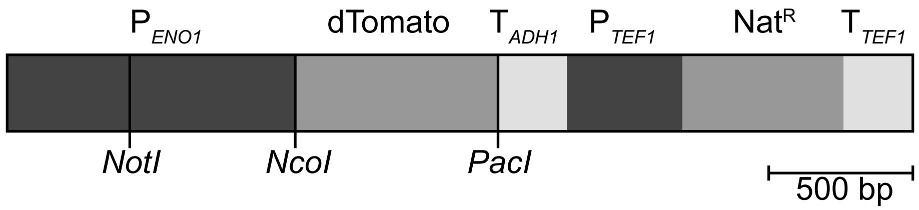

B

```
ATGGTTTCAAAAGGTGAAGAAGTTATTAAAGAATTTATGAGATTTAAAGTTAGAATG-
GAAGGTTCAATGAATGGTCATGAATTTGAAATTGAAGGTGAAGGTGAAGGTAGACCATA
TGAAGGTACTCAAACTGCTAAATTAAAAGTTACTAAAGGTGGTCCATTACCATTTGCTT
GGGATATTTTATCACCACAATTTATGTATGGTTCAAAAGCTTATGTTAAACATCCAGCT
GATATTCCAGATTATAAAAAATTATCATTTCCAGAAGGTTTTAAATGGGAAAGAGTTAT
GAATTTTGAAGATGGTGGTTTAGTTACTGTTACTCAAGATTCATCATTACAAGATGGTA
CTTTAATTTATAAAGTTAAAATGAGAGGTACTAATTTTCCACCAGATGGTCCAGTTATG
CAAAAAAAAACTATGGGTTGGGAAGCTTCAACTGAAAGATTATATCCAAGAGATGGTGT
TTTAAAAGGTGAAATTCATCAAGCTTTGAAATTGAAAGATGGTGGTCATTATTTGGTTG
AATTTAAAACTATTTATATGGCTAAAAAACCAGTTCAATTGCCAGGTTATTATTATGTT
GATACTAAATTGGATATTACTTCACATAATGAAGATTATACTATTGTTGAACAATATGA
AAGATCAGAAGGTAGACATCATTTGTTTTTGTATGGTATGGATGAATTGTATAAATAA
```

Fig. S4. CUG-optimized dTomato. (A) The dTomato sequence (dTomato, grey box, based on GenBank sequence AY678268) was codon optimized for CUG-clade organisms and synthesized (Genscript Inc., Piscataway, NJ). Its expression is under the control of the constitutive ENO1 C. albicans promoter (P_{ENO1}, dark grey box, 1kb upstream of ORF19.395 from Assembly 21 at www.candidagenome.org) and the TEF1 terminator (T_{TEF1}, GenBank sequence S78175.1). The transformation plasmid contains the nourseothricin resistance gene (Nat^R, grey box, GenBank sequence AY854370.1) under the constitutive TEF1 promoter (P_{TEF1}, dark grey box, GenBank sequence S78175.1) and with the ADH1 terminator (T_{ADH1}, GenBank sequence EU493339.1) for selection purposes. The NotI restriction enzyme was used to stimulate homologous recombination at the ENO1 locus in C. albicans. (B) DNA sequence of codon-optimized dTomato gene (Shaner et al., 2004).

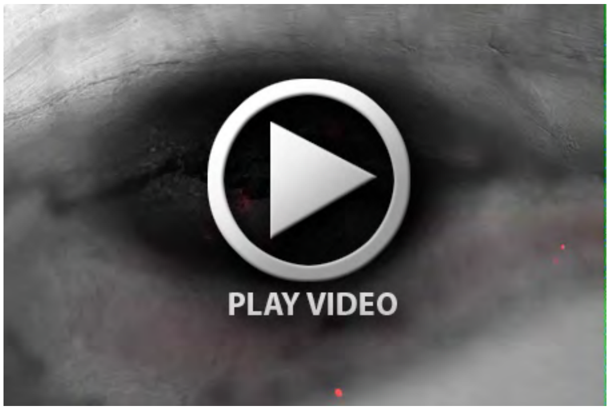

Movie 1. *C. albicans* swimbladder infection is limited to the epithelium. Animated Z-stack of image shown in Fig. 1D. Yeast and filaments are observed at the interface with the epithelium of the swimbladder.

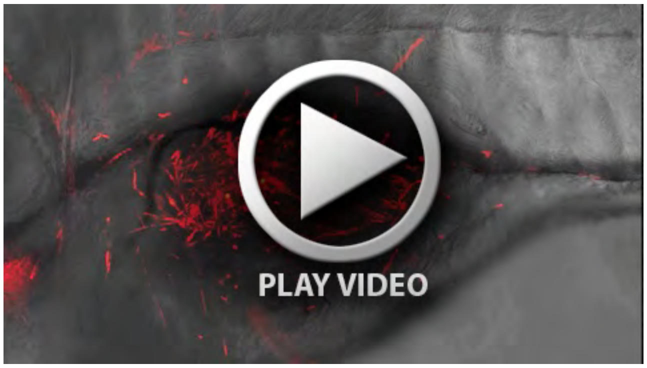

Movie 2. Invasion of surrounding tissue by *C. albicans* filaments emanating from the swimbladder. FITC-labeled CAF2-dTomato yeast were used for infection. Filaments derived from these inoculating yeast are dTomato-expressing but not FITC labeled, as new cell wall does not inherit the FITC fluorescence. Animated Z-stack of image shown in Fig. S1.

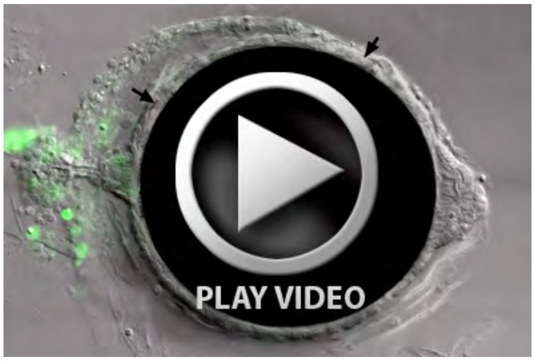

Movie 3. *C. albicans* contained within a phagocyte do not elicit EGFP fluorescence in surrounding epithelial cells (black arrows). Animated Z-stack of image shown in Fig. 3C.

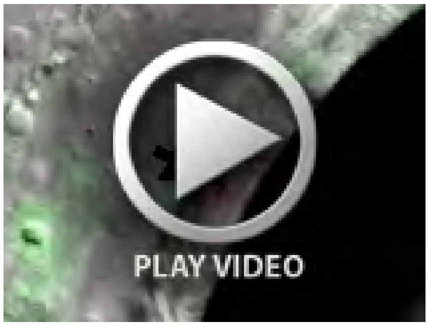

Movie 4. Magnification of Movie 3. Animated, zoomed Z-stack of image shown in Fig. 3C. Black arrow indicates focus of infection with *C. albicans* within a phagocyte and the surrounding epithelial cells expressing minimal green fluorescence.

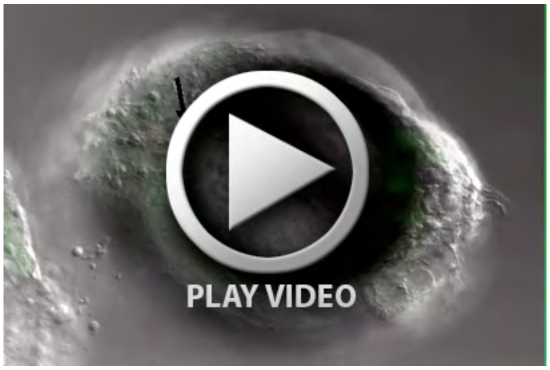

Movie 5. *C. albicans* not contained by phagocytes elicit EGFP expression at the foci of infection in adjacent epithelial cells (black arrow). Animated Z-stack of image shown in Fig. 3E.

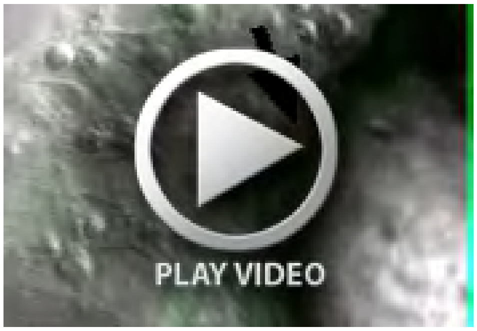

Movie 6. Magnification of Movie 5. Animated Z-stack of image shown in Fig. 3E. Black arrow indicates EGFP fluorescence in cells surrounding *C. albicans*.

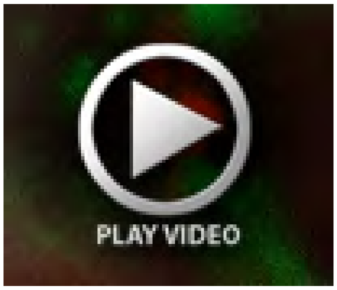

Movie 7. Neutrophil (green fluorescence) in direct contact with *C. albicans* (red fluorescence). Animated Z-stack of image shown in Fig. 5B, right panel.

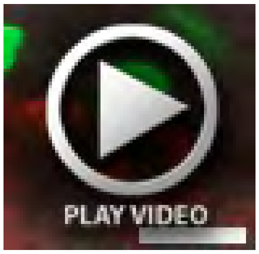

Movie 8. Neutrophil (green fluorescence) in direct contact with *C. albicans* (red fluorescence). Animated Z-stack of image shown in Fig. 5A, left panel.
